# Predictive Biomarkers for Immunotherapy in Gastric Cancer: Current Status and Emerging Prospects

**DOI:** 10.3390/ijms242015321

**Published:** 2023-10-18

**Authors:** Wanting Hou, Yaqin Zhao, Hong Zhu

**Affiliations:** 1 Division of Abdominal Tumor Multimodality Treatment, Cancer Center, West China Hospital, Sichuan University, Chengdu 610065, China; houwanting@wchscu.cn (W.H.); zhaoyaqin@163.com (Y.Z.); 2 Department of Radiation Oncology, Cancer Center, West China Hospital, Sichuan University, Chengdu 610065, China

**Keywords:** gastric cancer, immune checkpoint inhibitor, immunotherapy, predictive biomarker

## Abstract

Gastric cancer presents substantial management challenges, and the advent of immunotherapy has ignited renewed hope among patients. Nevertheless, a significant proportion of patients do not respond to immunotherapy, and adverse events associated with immunotherapy also occur on occasion, underscoring the imperative to identify suitable candidates for treatment. Several biomarkers, including programmed death ligand-1 expression, tumor mutation burden, mismatch repair status, Epstein–Barr Virus infection, circulating tumor DNA, and tumor-infiltrating lymphocytes, have demonstrated potential in predicting the effectiveness of immunotherapy in gastric cancer. However, the quest for the optimal predictive biomarker for gastric cancer immunotherapy remains challenging, as each biomarker carries its own limitations. Recently, multi-omics technologies have emerged as promising platforms for discovering novel biomarkers that may help in selecting gastric cancer patients likely to respond to immunotherapy. The identification of reliable predictive biomarkers for immunotherapy in gastric cancer holds the promise of enhancing patient selection and improving treatment outcomes. In this review, we aim to provide an overview of clinically established biomarkers of immunotherapy in gastric cancer. Additionally, we introduce newly reported biomarkers based on multi-omics studies in the context of gastric cancer immunotherapy, thereby contributing to the ongoing efforts to refine patient stratification and treatment strategies.

## 1. Introduction

Gastric cancer (GC) presents a significant global health concern, ranking fifth in incidence and fourth in mortality worldwide. In 2020 alone, there were over one million new GC cases, resulting in approximately 769,000 deaths [1]. Advanced GC (AGC), also known as mid- to late-stage gastric cancer, refers to tumors that invade the muscular layer or all layers of the stomach. Previously, AGC patients had limited treatment options, primarily relying on chemotherapy with modest efficacy. In recent years, immunotherapy utilizing immune checkpoint inhibitors (ICIs) has shown therapeutic effectiveness in specific GC patients, especially those exhibiting specific traits, such as the high expression of programmed death ligand-1 (PD-L1), high microsatellite instability (MSI-H), and patients who are Epstein–Barr Virus (EBV)-positive. These patients have shown a higher response rate to immunotherapy [2]. The US Food and Drug Administration (FDA) now approves pembrolizumab as a third-line treatment for recurrent or metastatic GC or gastroesophageal junction adenocarcinoma with PD-L1 expression ≥ 1%. Additionally, nivolumab in combination with chemotherapy has been approved as a first-line therapy for advanced or metastatic GC and gastroesophageal junction cancer (GEJC), irrespective of PD-L1 expression. For human epidermal growth factor receptor 2 (HER2)-positive AGC, the FDA recommends a combination of programmed death-1 (PD-1) monoclonal antibody (pembrolizumab) with HER-2 monoclonal antibody (trastuzumab) along with chemotherapy. Nevertheless, a considerable number of patients continue to demonstrate an inability to benefit from immunotherapy, and immunotherapy-related adverse reactions are observed intermittently. Further research into immune-related biomarkers for GC becomes especially crucial for the precise selection of suitable candidates for immunotherapy.

Advancements in genetic testing technology have led to the identification of specific molecular markers for GC, recognizing it as a heterogeneous disease. The Cancer Genome Atlas (TCGA) project conducted a comprehensive evaluation in 2014, classifying GC into four distinct subtypes: EBV-positive GC, MSI GC, genomically stable (GS) GC, and GC with chromosomal instability (CIN) [3]. The Asian Cancer Research Group (ACRG) proposed an additional molecular typing of GC based on samples from Asian populations, further correlating it with tumor prognosis. ACRG classified GC into four subtypes: the MSI subtype, predominantly Lauren intestinal type with early clinical staging (stage I or II) and the best prognosis; MSS/EMT subtype, displaying a significantly lower age of onset than other subtypes, with the absent expression of cell adhesion gene CDH1, mostly corresponding to Lauren diffuse type, late staging (stage III or IV), and the worst prognosis among the four subtypes; MSS/TP53+ subtype, with a higher frequency of EBV infection; and the MSS/TP53- subtype, enriched with HER-2 gene amplifications [4].

While these molecular subtypes have provided some guidance in treating and predicting outcomes for GC patients, more effective predictive biomarkers, particularly for populations benefiting from immunotherapy response, are needed. Currently, HER2 and PD-L1 expression are the sole predictors guiding treatment choice in AGC patients. Targeting HER2 has shown significant improvements in overall survival for HER2-positive patients [5]. However, PD-L1 expression’s relationship with GC response remains controversial [6]. Other potential predictive biomarkers for immunotherapy response in GC include MMR status, TMB, and EBV infection, but larger-scale studies are necessary to validate their effectiveness [7]. Liquid biopsy and emerging technologies like multiplex immunohistochemistry (mIHC) and single-cell sequencing offer new tools for individualized GC research [8,9,10]. Given the potential value of immunotherapy in GC treatment and the occurrence of adverse reactions associated with immunotherapy, identifying patients who may benefit from this treatment is of paramount importance. Thus, in this review we aim to summarize current clinically relevant biomarkers predicting GC immunotherapy prognosis and identifying beneficial patient populations. Additionally, we will introduce new predictive biomarkers of GC immunotherapy discovered using novel technologies. We hope this review will pave the way for new directions in GC immunotherapy biomarker research.

## 2. Predictive Biomarkers for Immunotherapy in Gastric Cancer

### 2.1. PD-L1 Expression as the Biomarker for GC Immunotherapy

**Figure 1:**
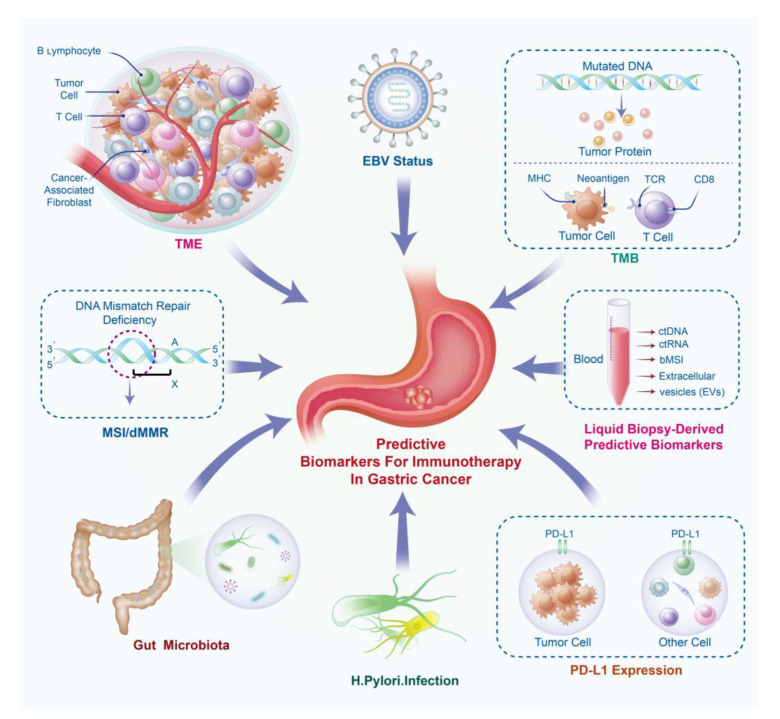
Predictive biomarkers of gastric cancer immunotherapy. bTMB, blood total mutation burden; bMSI, blood microsatellite instability; ctDNA, circulating tumor DNA; ctRNA: circulating tumor RNA; dMMR: mismatch repair deficiency; EBV, Epstein–Barr Virus; MSI, microsatellite instability; PD-L1, programmed cell death-ligand 1; TCR, T cell receptor; TMB, total mutation burden; TME, tumor microenvironment.

PD-L1 is a critical ligand protein expressed on tumor cells and tumor-associated antigen-presenting cells. Its binding to programmed death-1 (PD-1) on T cells leads to T cell deactivation, enabling tumor cells to evade the host’s immune response. ICIs targeting PD-L1 or PD-1 can interrupt this interaction, activating T-cell anti-tumor immunity [11]. Consequently, PD-L1 expression levels serve as a crucial evaluation index for ICI response [12] (Figure 1).

Immunohistochemistry (IHC) is the primary method for detecting PD-L1 protein expression levels. To standardize PD-L1 testing, the FDA has authorized four PD-L1 diagnostic monoclonal antibodies for IHC: 22C3, 28-8, SP142, and SP263. IHC indicators such as combined positive score (CPS) and tumor proportion score (TPS) are used to evaluate PD-L1 expression. TPS represents the percentage of tumor cells stained with any intensity of PD-L1, while CPS accounts for PD-L1-positive tumor cells and tumor-associated immune cells divided by the total number of tumor cells [13]. Several clinical studies have evaluated TPS or CPS as predictive biomarkers for ICI therapy in GC (Table 1).

In the third-line therapy setting, KEYNOTE-012 reported on the response of AGC patients to ICI treatment. The study evaluated the safety and efficacy of pembrolizumab in PD-L1-positive recurrent or metastatic GC or GEJC patients. The results demonstrated promising the anti-tumor activity and safety of pembrolizumab for PD-L1-positive GC/GEJC patients (TPS high) and highlighted the significance of measuring PD-L1 expression in mononuclear inflammatory cells [14]. KEYNOTE-059 investigated pembrolizumab as third-line therapy for GC or GEJC patients, showing an overall response rate (ORR) of 22.7% for patients with CPS ≥ 1, while PD-L1-negative tumors had an ORR of 8.6%. The study also explored the predictive value of the MSI status and immune-related gene expression for immunotherapy [15]. Subsequently, based on the KEYNOTE-059 results, the FDA granted the accelerated approval of pembrolizumab for patients with recurrent locally advanced or metastatic GC or GEJC with CPS ≥ 1. The CheckMate032 study evaluated dual immune checkpoint inhibitors, combining nivolumab with ipilimumab, for metastatic esophagogastric cancers. The study demonstrated clinically meaningful antitumor activity for nivolumab and dual immune checkpoint inhibitors, irrespective of TPS and MSI status [16]. Post hoc exploratory analyses of CheckMate032 indicated that CPS with cutoffs of ≥5 and ≥10 were more accurate predictors of immunotherapy response than TPS. Additionally, inflammatory gene signatures were associated with the response to ICI treatment [17]. The ATTRACTION-2 trial showed nivolumab provided survival benefits to GC or GEJC patients, independent of TPS [18]. Meanwhile, in a subset analysis of the ATTRACTION-2 study, the TPS, blood-neutrophil-to-lymphocyte ratio (NLR), and serum Na levels were proposed as predictive biomarkers for the response to nivolumab in previously treated AGC patients [19]. JAVELIN Gastric 300 trial evaluated avelumab in third-line therapy for advanced GC/GEJC patients, but it did not improve overall survival (OS) or progress free survival (PFS) compared to chemotherapy. Subgroup analysis based on TPS ≥ 1 did not reveal significant differences between avelumab and chemotherapy arms in terms of OS [20]. The maintenance therapy study, JAVELIN Gastric 100, involving avelumab, revealed no significant improvement in OS when compared to chemotherapy. This was observed among patients with advanced GC or GEJC, both in the overall group and specifically within the population with positive PD-L1 expression (CPS ≥ 1) [21]. 

The KEYNOTE-061 trial assessed anti-PD-L1 therapy as a second-line treatment for AGC. Initial results indicated that pembrolizumab did not significantly enhance OS compared to paclitaxel in patients with a CPS ≥ 1 [22]. However, subsequent 2-year follow-up data unveiled encouraging trends, displaying the potential for enhanced OS with pembrolizumab when compared to paclitaxel within the CPS ≥ 1 subgroup. The 24-month OS percentages stood at 19.9% for pembrolizumab and 8.5% for paclitaxel. Notably, the efficacy of pembrolizumab in OS was more pronounced with PD-L1 enrichment: CPS ≥ 5 (24-month rate: 24.2% vs. 8.8%) and CPS ≥ 10 (24-month rate: 32.1% vs. 10.9%). However, no substantial differences in median PFS were observed across the treatment groups [47]. Additionally, a recent report involving exploratory analysis within the KEYNOTE-061 trial utilized RNA sequencing data to evaluate the 18-gene T-cell-inflamed gene expression profile (TcellinfGEP) and ten non-TcellinfGEP signatures. This analysis revealed that TcellinfGEP exhibited associations with ORR and PFS specifically for pembrolizumab, without the same effects for paclitaxel. Notably, the TcellinfGEP-adjusted mMDSC signature exhibited adverse relationships with ORR, PFS, and OS for pembrolizumab, whereas such associations were absent in the case of paclitaxel [40]. In a phase I/II trial (UMIN000025947) investigating second-line therapy for AGC, a combination of nivolumab, paclitaxel, and ramucirumab was studied. This trial reported a median OS of 13.8 months for CPS ≥ 1 patient and 8.0 months for CPS < 1 patient [23]. Furthermore, a phase II trial conducted by the Arbeitsgemeinschaft Internistische Onkologie (AIO) evaluated the effectiveness of combining avelumab, ramucirumab, and paclitaxel as second-line therapy for GEJC patients. The trial findings indicated a median OS of 9.4 months for CPS < 5 patients compared to 14.0 months for CPS ≥ 5 patients, underscoring the prognostic value of CPS in predicting the response to immunotherapy in GC patients [24]. In the MAHOGANY study, an exploration was conducted involving the amalgamation of the anti-HER2 monoclonal antibody (margetuximab) and the anti-PD-1 monoclonal antibody (pembrolizumab) in individuals afflicted by trastuzumab-resistant HER2-positive gastro-esophageal adenocarcinoma. Among patients with positive PD-L1 expression, the observed ORR was 33%; in contrast, patients with negative PD-L1 expression displayed a markedly lower ORR of 7% [25].

In April 2021, the FDA approved nivolumab as a first-line treatment for advanced or metastatic GC patients, regardless of their PD-L1 expression. This decision was based on the findings of CheckMate 649 [26], which demonstrated significant improvements in OS and PFS for patients with a CPS of ≥1 and for all randomized patients with PD-L1 expression. However, although the combination of nivolumab and chemotherapy led to significant OS and PFS benefits for patients with a CPS of ≥5, long-term results for nivolumab plus ipilimumab versus chemotherapy did not reach statistical significance [48]. In the ORIENT-16 trial, the efficacy of sintilimab (PD-1 inhibitor) combined with chemotherapy for advanced GC/GEJC patients was studied. OS benefits were consistent across CPS cutoffs (CPS ≥ 1, 5, and 10) [27]. The KEYNOTE-062 study enrolled untreated, locally advanced/unresectable or metastatic G/GEJC patients with CPS ≥ 1. The result showed pembrolizumab, either alone or in combination with chemotherapy, did not show superiority in OS and PFS compared to chemotherapy alone [28]. In the context of first-line therapy for patients with HER2-negative, unresectable advanced or recurrent GC or GEJC, the ATTRACTION-4 study investigated the comparative efficacy between nivolumab plus chemotherapy and placebo plus chemotherapy. The results indicated that nivolumab plus chemotherapy significantly improved PFS, but not OS, with no significant prognostic difference observed between patients with a TPS ≥ 1 and those with a TPS < 1 [29]. In the KEYNOTE-659 study, researchers evaluated pembrolizumab in combination with chemotherapy in Japanese advanced GC/GEJC patients who were PD-L1-positive (CPS ≥ 1) and HER2-negative. Exploratory analysis of OS according to CPS status, in cohort 1 (pembrolizumab with S-1 + oxaliplatin), revealed that patients possessing a CPS ≥ 10 exhibited an OS of 14.9 months (95% CI, 9.5–19.1), while those with a CPS < 10 demonstrated an OS of 17.7 months (95% CI, 13.4–23.7). Within cohort 2 (pembrolizumab with S-1  +  cisplatin), patients with CPS ≥ 10 experienced an OS of 15.5 months (95% CI, 11.5–22.9), whereas those with CPS < 10 showcased an OS of 21.7 months (95% CI, 8.3–NE). Similarly, the median PFS in cohort 1 was recorded at 8.1 months (95% CI, 5.5–12.6) for CPS ≥ 10 patients and 12.6 months (95% CI, 6.6–NE) for CPS < 10 patients. As for cohort 2, CPS ≥ 10 patients achieved a median PFS of 7.0 months (95% CI, 5.1–NE), and CPS < 10 patients reached a median PFS of 14.8 months (95% CI, 5.8–16.4). Overall, the combination of pembrolizumab and chemotherapy showcased a favorable combination of effectiveness and safety for patients diagnosed with PD-L1-positive, HER2-negative GC or GEJC [30].

Numerous studies have also explored immunotherapy’s potential as a neoadjuvant option for locally advanced GC. In a phase II exploratory trial (NCT03878472), the effectiveness of combining an ICI (camrelizumab), antiangiogenic agent (apatinib), and chemotherapy for neoadjuvant/conversion therapy in cT4a/bN+ GC was assessed. This study employed sequential multi-omics testing encompassing whole-exome sequencing (WES), transcriptome sequencing, and T cell receptor (TCR) sequencing. This extensive analysis unveiled potential biomarkers for assessing pathological responses and dynamic alterations in tumor immune microenvironments, and T cell receptor repertoires in the context of neoadjuvant immunotherapy. Correlations were established between pathological responses and MSI status, CPS, and TMB. Notably, potential biomarkers for pathological responses were identified, including RREB1 and SSPO mutations, immune-related signatures, and a peripheral T cell expansion score [32]. In the Neo-PLANET phase II trial, neoadjuvant camrelizumab was examined alongside concurrent chemoradiotherapy for locally advanced GC/GEJC. Interestingly, the study discovered that the pathological complete response (pCR) rate in PD-L1-positive tumors (defined by CPS at 1, 5, or 10 cutoffs) did not significantly differ from PD-L1-negative tumors. However, analysis of somatic mutations through the WES of treatment samples revealed a notably higher pCR rate in patients with a pretreatment TMB ≥ median level (4.04 mutations/Mb) than those with TMB < median level [33]. Additionally, a phase 2 clinical trial (NCT04065282) investigated the efficacy and safety of neoadjuvant sintilimab, oxaliplatin, and capecitabine in patients with locally advanced and resectable GC or GEJC. The patients were stratified based on their CPS levels (CPS ≥ 1, CPS ≥ 5, and CPS ≥ 10). The outcomes revealed that the combination of sintilimab with oxaliplatin and capecitabine demonstrated promising effectiveness, characterized by an encouraging rate of pathologic complete response (pCR), along with a favorable safety profile within the neoadjuvant context. These findings provide support for CPS as a predictive biomarker, aiding in the identification of patients likely to benefits from neoadjuvant anti-PD-1 treatment. Within the subgroup of patients with CPS < 1, pCR and major pathological response (MPR) rates stood at 9.1% and 27.3%, respectively. In contrast, among patients with CPS ≥ 1, the corresponding rates were 28.6% for pCR and 57.1% for MPR. It is worth noting that in the CPS ≥ 5 group, pCR and MPR rates were 27.3% and 54.5%, respectively. Among patients with CPS ≥ 10, pCR and MPR rates reached 33.3% and 50.0%, respectively [34].

The role of PD-L1 expression in GC immunotherapy’s significance is a topic of ongoing debate. Data from the CheckMate-649, KEYNOTE-062, and KEYNOTE-590 trials reveal that combining ICI with chemotherapy does not confer benefits for AGC patients with low PD-L1 expression (CPS 1-4 in CheckMate-649 and CPS 1-9 in KEYNOTE-062) [49]. Furthermore, a systematic review noted that TPS was the primary predictor of ICI benefit in squamous cell carcinoma patients, whereas CPS emerged as the strongest predictor for AGC [50]. A meta-analysis demonstrated favorable OS and PFS when ICIs were combined with first-line chemotherapy for GC/GEJC patients, irrespective of CPS status [51]. Another meta-analysis proposed CPS ≥ 1 as the threshold for ICI monotherapy’s survival advantage [52]. A thorough analysis incorporating CPS ≥ 10 GC patients’ data from KEYNOTE-059, KEYNOTE-061, and KEYNOTE-062 consistently showcased improved clinical outcomes with pembrolizumab across treatment lines [53]. Similarly, a multicenter biomarker cohort study of nivolumab treatment for GC identified CPS and MSI as independent yet valuable biomarkers for nivolumab response [54].

The reasons why PD-L1 cannot effectively predict the efficacy of immunotherapy may include the following:

1. Tumor heterogeneity: PD-L1 expression can vary within the tumor and between different tumor sites, leading to inconsistent results when assessing its predictive value. Measuring PD-L1 on circulating tumor cells (CTCs) may address heterogeneity, but its consistency with tumor cell PD-L1 remains uncertain [8].
2. Dynamic nature of PD-L1 expression: PD-L1 expression levels can change over time, making a single assessment unreliable for predicting long-term treatment outcomes. To enhance PD-L1’s predictive value, researchers suggest using multiple biopsies for improved accuracy [55].
3. Immune microenvironment: the presence of other immune cells and their interactions within the tumor microenvironment can influence treatment response independently of PD-L1 expression [56].
4. Immunotherapy mechanisms: immunotherapies may work through multiple mechanisms; some patients may respond even if PD-L1 expression is low or absent [57].
5. Sample collection and testing methods: variability in sample collection and testing methods can affect the accuracy of PD-L1 assessment [58].
6. Patient-specific factors: patient-specific factors, such as their overall health, prior treatments, and individual immune profiles, can influence treatment response independently of PD-L1 status [59].

Thus, due to these complexities, PD-L1 expression alone may not be sufficient to reliably predict the effectiveness of immunotherapy, and a more comprehensive approach considering multiple factors is often necessary.

### 2.2. Microsatellite Instability Status and Defective Mismatch Repair as the Biomarker for GC Immunotherapy

MSI-H or defective mismatch repair (dMMR) has emerged as a significant biomarker for predicting immunotherapy response in various solid tumors. In 2017, the FDA approved pembrolizumab for treating unresectable or metastatic solid tumors with MSI-H/dMMR status [60]. And in the case of MSI-H/dMMR colon cancer patients, pembrolizumab has been employed as a first-line therapy. The dysregulated expression of MMR genes can impair cellular repair function during DNA replication, leading to MSI. The accumulation of mutations in tumor cells caused by a loss of MMR gene function is thought to make them more vulnerable to recognition by immune cells, resulting in a favorable response to immunotherapy [61].

MSI-H/dMMR is a significant molecular subtype of GC [3,4]. This phenotype is present in approximately 10% of GC and GEJC patients, and it is more commonly detected in early-stage GC and elderly patients aged over 85 years [62]. Several clinical studies have examined the response of MSI-H/dMMR GC/GEJC patients to ICI, as presented in Table 1. In the KEYNOTE-158 trial, which enrolled patients with MSI-H solid tumors, 51 dMMR/MSI-H GC patients exhibited a sustained response to pembrolizumab, with an ORR of 31.0% and a median OS of 11.0 months [37]. Similarly, in the KEYNOTE-059 trial, MSI-H/dMMR GC patients showed a higher ORR of 57.1% compared to 9% in MSS/pMMR GC patients [15]. Moreover, neoadjuvant treatment utilizing nivolumab combined with ipilimumab yielded a notably high rate of pCR, reaching 59%, among patients with resectable dMMR/MSI-H GC or GEJC [36]. Subsequent to a post hoc analysis of the KEYNOTE-059, KEYNOTE-061, and KEYNOTE-062 trials, it was revealed that both pembrolizumab as monotherapy and the combination of pembrolizumab with chemotherapy displayed enduring anti-tumor effects in patients with advanced dMMR/MSI-H GC [63]. A comprehensive meta-analysis encompassing randomized clinical trials such as KEYNOTE-062, CheckMate-649, JAVELIN Gastric 100, and KEYNOTE-061 reported that within a cohort of 123 patients diagnosed with MSI-H gastric cancer, the hazard ratio (HR) for overall survival advantage using anti-PD-1-based treatments was 0.34 for MSI-H individuals, in comparison to 0.85 for those with MSS status [64]. 

Despite these findings, the current sample size is relatively small to establish MSI-H/dMMR as a direct predictor of immunotherapy effectiveness in these patients, given the relatively lower incidence of MSI-H GC [65]. IHC and polymerase chain reaction (PCR) are the commonly used methods for detecting MSI status [66]; however, MSI status exhibits heterogeneity [67], which can be influenced by multiple factors such as race, sampling factors, antibodies used, and subjective judgment by pathologists [68]. Moreover, MSI status as a marker for immunotherapy response is only a rough indicator, as approximately 50% of MSI-H tumor patients demonstrate inherent resistance to PD-1 inhibitors. Therefore, further exploration of key immune response indicators in MSI-H patients is necessary. For example, in this context, Kwon et al. proposed that the T-cell receptor repertoire was associated with longer progression-free survival to pembrolizumab, and an increase in PD-1 + CD8 + T cells correlated with durable clinical benefit in MSI-H GC patients [69].

### 2.3. Tumor Mutation Burden (TMB) as the Biomarker for GC Immunotherapy

Tumor mutation burden (TMB) is a commonly used metric to quantify the number of mutations per megabase (Mb) of genomic DNA sequencing in tumors. This measurement represents the count of nonsynonymous mutations identified in the coding regions of a tumor genome. Tumors with a high TMB (TMB-H) are believed to have a greater number of neoantigens, which can potentially be recognized and targeted by the immune system. Consequently, patients with high-TMB tumors are considered more likely to benefit from immunotherapy treatments [70]. Notably, in 2020, pembrolizumab monotherapy received accelerated FDA approval for previously treated, unresectable/metastatic TMB-H solid tumors (defined as TMB ≥ 10 mutations/Mb), based on the KEYNOTE-158 trial results. However, recent studies have raised concerns about relying solely on TMB-H as the predictive biomarker for tumor immunotherapy response [71,72]. Several studies have also explored the potential of high TMB as a predictive biomarker in immunotherapy for gastric cancer (GC), as presented in Table 1.

In a phase Ib/II clinical trial (NCT02915432), the safety and efficacy of toripalimab, a humanized PD-1 antibody, were evaluated in patients with AGC. The study investigated the potential of PD-L1 expression and TMB as predictive biomarkers for toripalimab response. The results showed that patients with TMB-H (defined as TMB ≥ 12 mutations/Mb) had significantly better OS compared to those with low TMB, while PD-L1 overexpression did not correlate with survival benefit [31]. An exploratory analysis of the KEYNOTE-061 trial revealed a strong association between TMB and the efficacy of second-line pembrolizumab in GC/GEJC patients. This study suggested that tissue TMB (tTMB) is a significant and independent predictor of pembrolizumab response, beyond PD-L1 status. Even after excluding patients with known MSI-H tumors, tTMB still showed a significant association with the pembrolizumab response [73]. In prespecified exploratory analyses of EPOC1706, which tested lenvatinib plus pembrolizumab in AGC patients either in first-line or second-line therapeutic settings, objective responses were achieved by 84% of patients with CPS ≥ 1 and 40% of patients with CPS < 1. In the subgroup of patients with CPS ≥ 10, 100% had an objective response. For patients with high TMB (TMB ≥ 10), the objective response rate was 82%, and for patients with low TMB (TMB < 10), the objective response rate was 60%. The median progression-free survival was 9.1 months in patients with CPS ≥ 1 and 5.9 months in patients with CPS < 1. The median progression-free survival was 9.8 months for patients exhibiting high TMB, while those with low TMB registered a slightly shorter median PFS of 9.5 months [38]. Notably, within the exploratory analysis of the KEYNOTE-062 clinical trial, an evident association emerged between TMB levels and the clinical effectiveness of first-line pembrolizumab in combination with chemotherapy among AGC patients. However, it is pertinent to highlight that the predictive prowess of TMB underwent attenuation upon the exclusion of patients with MSI-H tumors [41]. 

TMB’s reliability is limited by several factors. Firstly, TMB only represents the mutation burden, but not all mutations generate new antigens. Therefore, TMB can only provide indirect evidence for predicting and assessing the generation of new antigens [71]. Additionally, TMB is influenced by various factors such as tumor type, genetic alterations in the tumor microenvironment, exposure to external carcinogens, and detection methods. These factors can lead to TMB heterogeneity, which affects its accuracy as a predictive biomarker. TMB detection is also impacted by sample quality, detection methods, and analytical techniques. While WES is considered the gold standard for TMB detection, its high cost, time-consuming nature, and requirement for fresh specimens limit its use. Targeted sequencing panels offer a promising alternative to WES, allowing simultaneous analysis of multiple molecular indicators in addition to TMB, but they also require high sample purity [74]. Currently, researchers are exploring the combination of TMB with other molecular indicators for screening and predicting the efficacy of immunotherapy in GC. For example, the study of Chida et al. proposed low TMB as a negative predictor of PD-1 blockade responses in MSI-H/dMMR gastrointestinal tumor patients [75], and Wang et al. suggested using the combination of blood MSI and blood TMB to screen GC patients for immunotherapy benefit [76]. 

### 2.4. Epstein–Barr Virus Status as the Biomarker for GC Immunotherapy

Epstein–Barr Virus (EBV) is a common human herpesvirus that infects over 90% of the population and has been associated with various malignancies, including GC [77]. EBV-associated GC (EBVaGC) is a distinct subtype, accounting for approximately 9% of all GC cases. It exhibits unique features, such as occurring in younger patients, being more prevalent in males, primarily localizing in the upper part of the stomach, showing marked immune cell infiltration, and having a favorable prognosis [3,78]. EBVaGC patients also display distinct immune characteristics, including changes in immune response genes, elevated PD-L1 expression in both cancer and immune cells, increased T and NK cell infiltration, heightened expression of immune checkpoint markers, and elevated levels of some anti-tumor immunity factors [79,80,81]. As a result, EBV infection has been considered a positive factor for GC immunotherapy.

Some studies have indicated a positive correlation between EBVaGC and immunotherapy response (Table 1). In a prospective phase 2 clinical trial, all EBV-positive GC patients treated with pembrolizumab achieved a partial response (PR), with a longer median duration of response of 8.5 months [39]. In another prospective observational study by Xie et al., nine patients with stage-IV EBVaGC were treated with ICIs, and three patients showed PR, while five patients had stable disease, all of whom displayed positive PD-L1 expression. This study suggested that combining EBV and PD-L1 might be a more accurate biomarker combination for determining the efficacy of immunotherapy in GC [42]. However, a subsequent single-arm, phase 2 prospective clinical trial enrolled six EBV-positive mGC patients treated with camrelizumab, but none of them achieved an objective response, raising doubts about EBV positivity as a reliable predictor for immunotherapy response in mGC [43]. Nakayama et al. found in their study that a high EBV copy number per genome (>10 copies) correlated with PD-L1 expression in tumor cells but poor disease survival in EBVaGC [82]. 

While EBV-positive is an important subtype of GC, and some EBV-positive AGC patients have shown a response to ICI, the predictive value of EBV positivity for ICI response in metastatic GC patients remains uncertain [83]. Large-scale studies are needed to establish the relationship between EBV infection and GC immunotherapy response. Some existing studies propose using EBV as an adjuvant predictor of immune response in GC patients [84]. For example, combining PD-L1 and CD8 could identify EBVaGCs with high immunoreactivity in patients with pMMR [85]. A real-world study by Yu et al. found that better ORR and PFS were observed only in EBVaGC patients when they were combined with CPS ≥ 1 [86]. Further research is necessary to validate these findings and explore the potential relationship between EBV status and immune status in GC patients.

The gold standard method for detecting EBV in tissue sections is the EBV-encoded RNA (EBER) in situ hybridization (ISH). However, the EBER probe used for ISH is usually expensive and requires a certain level of sensitivity. IHC is a less costly and more convenient test for EBV infection, which detects the LMP-1 membrane protein encoded by EBV. However, IHC cannot detect the location or transcriptional quantity of the virus. Novel test methods for EBV infection have also been proposed, such as the use of NGS for detection. These new methods not only improve the accuracy of EBV detection but also provide insights into the mechanism of EBV infection and the immunological characteristics of EBVGC, which are crucial for understanding the response of EBVaGC to immunotherapy. 

### 2.5. Liquid Biopsy-Derived Predictive Biomarkers for GC Immunotherapy 

Considering the temporal and spatial heterogeneity of GC, liquid biopsy-based blood predictive biomarkers have emerged as a promising approach for predicting immunotherapy response. Liquid biopsy offers several advantages, such as being safe, convenient, and repeatable, allowing the real-time monitoring of disease progression and treatment response. It can also detect low levels of tumor cells, enhancing sensitivity and accuracy. Among the extensively investigated biomarkers in liquid biopsy for GC are circulating tumor cells (CTCs), circulating tumor DNA (ctDNA), and extracellular vesicles [8].

Various studies have emphasized the crucial role of liquid biopsy-based blood biomarkers in predicting and monitoring immunotherapy response in GC (Table 1). For instance, Ishiba et al. analyzed PD-L1 expression in circulating tumor RNA (ctRNA) from various cancer types, including GC. They found a high degree of concordance between PD-L1 protein expression in tumor tissues and PD-L1 gene expression in plasma, proposing PD-L1 expression in ctRNA as a viable assay for predicting and monitoring immunotherapy response [87]. Similarly, Jin et al. utilized NGS testing to conclude that dynamic ctDNA could serve as a potential biomarker for immunotherapy response in advanced GC, with decreasing ctDNA correlating to a higher response to treatment [88]. Zhang et al. revealed actionable alterations for targeted and immune therapy in Chinese AGC patients using molecular characterization with ctDNA [89]. Yue et al. demonstrated that measuring the abundance of PD-L1-high CTCs at baseline and monitoring their dynamic changes could indicate early therapeutic response for solid tumor patients [90]. 

Extracellular vesicles (EVs) have emerged as promising plasma components for monitoring immunotherapeutic outcomes in GC. Studies have reported a correlation between extracellular vesicle PD-L1 expression and immunotherapy response in GC. For instance, a study on AGC patients treated with nivolumab found that baseline soluble PD-L1 levels had the potential to predict survival [44]. Zhang et al. identified four plasma EV-derived proteins (ARG1/CD3/PD-L1/PD-L2) as an EV-score that robustly predicts immunotherapeutic outcomes at baseline and monitors disease progression with ICI treatment in GC patients [91]. In a phase Ib/2 trial, elevated plasma levels of PD-L1-expressing EVs were significantly associated with higher pCR among GEJC patients with PD-L1 CPS < 10 [35]. 

Despite its advantages, liquid biopsy as a screening method for immunotherapy predictive biomarkers in GC also has limitations. Firstly, biomarkers in blood are often not specific to the tumor, leading to possible false-positive results. Secondly, liquid biopsy may have sensitivity limitations and may not detect low concentrations of tumor biomarkers or DNA sequences. Additionally, different techniques and methods may yield varying results, and sample quality and processing can impact the outcomes, leading to technical limitations [92]. Therefore, further improvements are necessary to enhance the reliability of liquid biopsy for screening immune therapy biomarkers in GC, and more research is needed to address these issues. Furthermore, liquid biopsy can be used as a supplementary detection method in combination with other biomarkers to predict immune therapy responsiveness in GC.

### 2.6. Predictive Role of Gut Microbiota and Helicobacter pylori Infection in GC Immunotherapy

The gut microbiota plays a crucial role in maintaining overall health and influencing disease progression, including tumorigenesis. Recent research has highlighted its association with tumor progression and therapeutic efficacy, and it has been proposed as a potential biomarker to predict outcomes of immunotherapy for solid tumors [93,94]. Despite the known roles of certain gut microbiota in the development and immune regulation of GC, there still a lack of evidence on their potential as biomarkers for GC immunotherapy efficacy. Further research is needed to explore the value of gut microbiota in the context of GC immunotherapy.

One common infecting microorganism in GC patients is *Helicobacter pylori* (*H. pylori*). Its presence is closely associated with the development of GC, and *H. pylori*’s role in regulating the immune system has been proposed. Moreover, *H. pylori* can alter the immune microenvironment of GC, potentially impacting immunotherapy efficacy [95,96]. Study in mouse models of colon adenocarcinoma and melanoma have demonstrated the detrimental effect of *H. pylori* infection on immunotherapy [97]. Additionally, a retrospective analysis suggested an association between *H. pylori* infection and the outcome of immunotherapy for AGC patients [45]. However, this study had a limited sample size, and there is a lack of prospective studies on the prognostic implications of *H. pylori* in GC immunotherapy. Furthermore, the impact of *H. pylori* infection on immunity may vary significantly among individuals due to the complex infection process. Therefore, *H. pylori* infection cannot be used as the sole biomarker for population screening or predicting the efficacy of GC immunotherapy beneficiaries. Further research is necessary to better understand its role in the context of immunotherapy for GC.

### 2.7. Predictive Biomarkers of Tumor Microenvironment in GC Immunotherapy

The effectiveness of immunotherapy for tumors is directly influenced by the TME. Findings by Derks et al. demonstrated the heterogeneous nature of the tumor-immune microenvironment in GC, highlighting the pivotal role of TME in the immunotherapeutic response to GC [98]. 

Recent advances in omics technologies such as genomics, transcriptomics, and metabolomics have led to the identification of multiple TME-related biomarkers for GC immunotherapy. For example, Hu et al. employed a comprehensive multi-omics approach, including transcriptomics RNA-sequencing (mRNA, LncRNA, miRNA), DNA methylation, and gene mutations, to propose two distinct molecular subtypes (CS1 and CS2) of GC. They noted that the CS2 group exhibited higher immunocyte infiltration, suggesting potential immunotherapeutic benefits within this subgroup [99]. Fu et al. compared gene expression and immune markers between low- and high-TMB groups. They developed an immune prognostic model for GC based on their findings [100]. Yuan et al. integrated transcriptomics, proteomics, and metabolomics data, revealing HER2-associated metabolic heterogeneity in GC linked to responses to immunotherapy and neoadjuvant chemotherapy. Their study highlighted the quiescent and aspartate and glutamate subtypes as more likely to benefit from immunotherapy [101]. Wang et al. proposed a gene-based antigen processing and presentation signature (APscore) for prognostication and prediction of response to immune checkpoint inhibitors (ICIs) in AGC patients [102]. Shi et al. characterized glycometabolism and the tumor immune microenvironment to predict clinical outcomes in GC. They identified patients with a high Gluco-Immune Score as having potential for benefiting from immunotherapy [103]. Zeng et al. developed an open-source TMEscore R package, validated in a prospective phase 2 clinical trial of mGC patients treated with pembrolizumab, demonstrating the TME score’s superior accuracy over CPS, TMB, MSI, and EBV [104]. He et al. identified molecular features correlating with tumor immunity in GC, providing potential biomarkers for stratifying GC patients who are responsive to immunotherapy [105]. Chen et al. leveraged deep learning to propose immune subtypes and landscape analysis of GC based on whole-slide images, offering insights to improve immunotherapeutic strategies [106]. Cheong et al. established a 32-gene signature with prognostic and predictive capabilities for GC. They demonstrated that these molecular subtypes could forecast responses to adjuvant chemotherapy after gastrectomy and to immunotherapy in patients with metastatic or recurrent GC [107].

The efficacy of tumor immunotherapy is linked to immune cell quantity, their spatial relationships, and TME organization [108,109]. Tumor-infiltrating lymphocytes (TILs), comprising T cells, B cells, and NK cells, play a pivotal role in immune responses against tumors and are associated with immunotherapy efficacy [110]. The retrospective study of Tong et al. proposed intertumoral CD8+ TILs as a predictive factor for chemoimmunotherapy response in PD-L1-negative AGC [46]. Distinctive TME structures, like tertiary lymphoid structures (TLS), have also been correlated with positive immunotherapy outcomes [111]. TLS presence is also believed to predict responses to anti-PD1 therapy in GC [112]. Novel immune checkpoints like CD96 and CD73 have also been proposed as potential GC immunotherapy biomarkers. CD96 could guide precision medicine in adjuvant chemotherapy, immunotherapy, and targeted therapies [113]. High CD73 expression indicates better chemotherapeutic responsiveness but a poorer pembrolizumab response in GC [114]. 

mIHC and multiple immunofluorescence (mIF) are advanced techniques that significantly contribute to the understanding of TME immune status, offering insights into the immunological landscape. A meta-analysis across ten cancer types treated with ICI highlighted the superiority of mIHC/mIF over TMB, gene expression profiling, and PD-L1 expression biomarkers in predicting immunotherapy response [115]. In a recent study, Chen et al. employed mIHC and multi-dimensional analyses to predict immunotherapy response in GC. Their findings suggest that the TIL signature identified via mIHC holds promise as a prognostic biomarker for anti-PD-1/PD-L1 immunotherapy response and OS prediction [116]. 

These findings underscore multi-omics technologies’ role in understanding GC’s TME characteristics, enabling sensitive tumor-biomarker discovery. Yet, limited adoption of these techniques and reliance on small cohorts or database data constrain reliability. Further in-depth research is essential to uncover predictive TME biomarkers for GC immunotherapy.

### 2.8. Predictive Biomarkers of Immunotherapy-Related Adverse Events in GC

Tumor immunotherapy-related adverse events (irAEs) refer to adverse reactions associated with tumor immunotherapy. The mechanisms underlying the development of irAEs remain unclear at present. These adverse events are currently believed to arise from an exaggerated response of the immune system against normal tissues rather than being directly caused by the tumor itself. irAEs can affect multiple organ systems, including the skin, gastrointestinal tract, liver, and lungs, with the severity varying among individuals. Treatment typically involves the reduction in the immunotherapeutic agent’s dosage or temporary suspension of treatment to alleviate these adverse reactions [117]. In the context of GC immunotherapy, the incidence of irAEs is not infrequent. Moreover, severe irAEs possess the capacity to pose a threat to patients’ overall survival.

Several studies have suggested a potential association between irAEs and a more favorable response to immunotherapy in patients [118,119]. Additionally, research has demonstrated a correlation between the occurrence of irAEs following immunotherapy and improved survival outcomes in GC patients [120,121,122]. Furthermore, certain studies have identified potential biomarkers for predicting the occurrence of irAEs [123]. For example, Jiang et al. explored the utility of biomarkers for irAE prediction by focusing on EV-derived proteins. They collected dynamic plasma samples from 102 GC patients who received ICIs and screened two EV-derived proteins, namely, inducible T-cell co-stimulator (EV-ICOS) and indoleamine 2,3-dioxygenase 1 (EV-IDO1), to prognosticate the development of irAEs [124]. Additionally, Jin et al. found that GC patients with genetic alterations in the CEBPA, FGFR4, MET, or KMT2B genes exhibited an increased likelihood of experiencing irAEs [88].

While research on biomarkers related to adverse events in gastric cancer immunotherapy is currently limited, investigating biomarkers for irAEs holds significant promise. This research can aid in the identification of individuals at risk for irAEs, enabling the mitigation of adverse event risks associated with immunotherapy. Additionally, it can contribute to the precise selection of the most suitable candidates for GC immunotherapy, making it one of the crucial research directions in the field of GC immunotherapy.

## 3. Conclusions

In recent years, standard treatment approaches for GC patients, including neoadjuvant therapy and advanced-stage cases, have recommended the use of single-agent and combination immunotherapy. However, clinical outcomes have demonstrated that a significant proportion of GC patients do not experience the benefits of immunotherapy, and adverse events are not uncommon. Therefore, the precise selection of GC patients who could benefit from immunotherapy is of utmost importance. In this comprehensive review, we have summarized various immunotherapy efficacy assessment markers reported in current clinical studies. These findings suggest that PD-L1 expression levels remain a widely used clinical biomarker for evaluating immunotherapy efficacy in GC. However, further attention is warranted to refine the criteria for its selection, particularly considering the variations associated with standard treatment protocols, intertumoral heterogeneity, and detection methods. While MSI status, EBV infection, and TMB levels have shown promising roles as predictive factors in certain GC immunotherapy clinical trials, large-scale clinical research is still required for further validation. The advent of novel techniques such as liquid biopsy and multi-omics studies brings renewed hope to the investigation of predictive biomarkers for GC immunotherapy efficacy. In this context, the exploration of combinations of molecular markers and personalized testing emerges as a crucial research direction in the field of GC immunotherapy.

## Figures and Tables

**Table 1:** Summary of biomarkers for colon cancer immunotherapy in reported clinical trials.

Line	ID	Study Type	Cancer Type	Patient Selection	Number	ICI	Predictive Biomarker	Test Method	Outcome
Third line	KEYNOTE-012	phase 1b	PD-L1-positive AGC	PD-L1-positive patients	39	pembrolizumab	TPS; PD-L1 expression in mononuclear inflammatory cells mononuclear inflammatory cell density score (MIDS)	PD-L1 IHC 22C3 pharmDx assay	MIDS 1: ORR 25%; MIDS 2:ORR 12%; MIDS 3: ORR 44%; TPS 0%: ORR 24%; TPS (50–100%): ORR 33% [14]
	KEYNOTE-059	phase 2	AGC and GEJC	all	259	pembrolizumab	CPS; MSI status	CPS: PD-L1 IHC 22C3; MSI status: DNA mismatch repair across five mononucleotide repeat markers (NR21, NR24, BAT25, BAT26, MONO27) using DNA extracted from formalin-fixed, paraffin-embedded tumor samples and blood (normal control) using the MSI Analysis System	ORR: 22.7% (CPS ≥ 1) vs. 8.6% (PD-L1-negative); 57.1% MSI-H patients experienced an objective response; responders had a higher 18-gene T-cell-inflamed expression profiling score compared to non-responders [15]
	CheckMate 032	phase 3	metastatic GC and GEJC	all	160	nivolumab ± ipilimumab	TPS; MSI status	TPS: IHC MSI status: PCR–based assay on the basis of the Bethesda panel of mononucleotide and dinucleotide markers	Responses were observed regardless of tumor PD-L1 status and MSI-status [16]
	In post hoc exploratory analyses from CheckMate 032	phase 3	metastatic GC and GEJC	all	163	nivolumab ± ipilimumab	TPS; CPS; inflammatory gene signatures/transcripts	TPS/CPS: IHC (Dako PD-L1 IHC 28-8 pharmDx assay); inflammatory gene signatures/transcripts: RNA sequencing	CPS ≥ 5 ORR: 19%; CPS ≥ 10 ORR: 26%; TPS ≥ 5 ORR: 8%; TPS ≥ 10 ORR: 9%; multiple inflammatory gene signatures/transcripts, including a signature consisting of four genes (CD274, CD8A, LAG3, and STAT1), showed associations with response to NIVO ± IPI [17]
	ATTRACTION-2	phase 3	advanced GC and GEJC	all	493	nivolumab	TPS	TPS:IHC 28-8 pharmDx assay	The survival benefit with nivolumab was independent of TPS [18]
	subset analysis of ATTRACTION-2 phase III trial	phase 3	AGC	all	45	nivolumab	PD-L1; MSI; EBV; TMB; NLR and Serum Na	PD-L1 IHC 22C3 pharmDx assay; MSI:IHC; EBV:ISH; TMB:NGS; NLR and Serum Na	In the nivolumab group, PD-L1 (+), low NLR, and normal Na (≥135 mmol/L) were associated with higher response and disease control rates, while tumor EBV infection and TMB were not [19]
	JAVELIN Gastric 300	phase 3	advanced GC and GEJC	all	371	avelumab	TPS	TPS: IHC	According to tumor PD-L1 expression ≥ 1%, no significant OS/PFS differences between the avelumab and chemotherapy arms [20]
Maintenance therapy	JAVELIN Gastric 100	phase 3	advanced GC and GEJC	all	805	avelumab	CPS	CPS: PD-L1 IHC 22C3	CPS ≥ 1 median OS was 14.9 months with avelumab versus 11.6 months with chemotherapy [21]
Second line	KEYNOTE-061	phase 3	advanced GC and GEJC	all	592	pembrolizumab	CPS	CPS: PD-L1 IHC 22C3	24-month OS (pembrolizumab vs. paclitaxel) was 19.9% vs. 8.5% (CPS ≥ 1), 24.2% vs. 8.8% (CPS ≥ 5), and 32.1% vs. 10.9% (CPS ≥ 10) [22]
	UMIN-CTR (UMIN000025947)	phase 1/2	AGC	all	43	nivolumab	CPS	CPS: PD-L1 IHC 22C3	Median survival time was 13.1 months (95% CI, 8.0–16.6 months): 13.8 months (95% CI, 8.0–19.5 months) in patients with CPS ≥ 1 and 8.0 months (95% CI, 4.8–24.1 months) in patients with CPS < 1 [23]
	Arbeitsgemeinschaft Internistische Onkologie (AIO)	phase 2	GEJC	all	60	avelumab	CPS	CPS: PD-L1 IHC	CPS < 5: mOS 9.4 mo (95% CI 7.2–11.2), CPS ≥ 5: mOS 14.0 mo (95% CI 12.8–15.3) [24]
	MAHOGANY	phase 1b-2	unresectable or metastatic gastroesophageal adenocarcinoma	HER2-positive, PD-L1-unselected	95	pembrolizumab	CPS	CPS: PD-L1 IHC 22C3	PD-L1-positive vs. PD-L1-negative ORR: 33% vs. 7% [25]
First line	CheckMate 649	phase 3	GC/GEJC	non-HER2-positive	1581	nivolumab	CPS; MSI status	CPS: IHC(Dako PD-L1 IHC 28-8 pharmDx assay)	Nivolumab-plus-chemotherapy resulted in significant improvements in OS and PFS versus chemotherapy in PD-L1 CPS ≥ 5 patients. Significant improvement in OS, along with PFS benefit, in PD-L1 CPS ≥ 1 and all-randomized patients Overall survival in patients with CPS ≥ 5 for nivolumab plus ipilimumab versus chemotherapy alone did not meet the prespecified boundary for significance [26]
	ORIENT-16	phase 3	advanced GC or GEJC	all	650	sintilimab	CPS	CPS: PD-L1 IHC 22C3	OS benefits were consistently observed at all pre-specified CPS cutoffs (CPS ≥ 1, 5, and 10) [27]
	KEYNOTE-062	phase 3	untreated, locally advanced/unresectable or metastatic GC or GEJC	CPS ≥ 1	763	pembrolizumab	CPS	CPS: IHC (Dako PD-L1 IHC 28-8 pharmDx assay)	Pembrolizumab prolonged OS vs. chemotherapy in patients with CPS ≥ 10 (median, 17.4 vs. 10.8 months), but this difference was not statistically tested. Pembrolizumab plus chemotherapy was not superior to chemotherapy for OS in patients with CPS ≥ 1 (12.5 vs. 11.1 months) or CPS ≥ 10 (12.3 vs. 10.8 months) or for PFS in patients with CPS ≥ 1 (6.9 vs. 6.4 months) [28]
	ATTRACTION-4	phase 2–3	untreated, unresectable advanced or recurrent GC or GEJC	HER2-negative	724	nivolumab	TPS	TPS: IHC 28-8 pharmDx kit	mOS: Nivolumab plus chemotherapy group: TPS Indeterminate or <1% 18.33 (15.74–21.13) vs. TPS ≥ 1%: 16.56 (10.48–22.67); mPFS: nivolumab plus chemotherapy group: TPS Indeterminate or <1% 11.30 (8.48–NR) vs. TPS ≥ 1%: 8.34 (4.27–12.45) [29]
	KEYNOTE-659	phase 2b	advanced GC or GEJC	PD-L1-positive, HER-2-negative	54	pembrolizumab	CPS	PD-L1 expression was centrally assessed during screening using the PD-L1 IHC 22C3 pharm assay	cohort 1 (pembrolizumab + S-1 + oxaliplatin): OS: 14.9 months (CPS ≥ 10) vs. 17.7 months (CPS < 10); cohort 2 (pembrolizumab + S-1 + cisplatin):OS:15.5 months (CPS ≥ 10) vs. 21.7 months (CPS < 10) [30]
	NCT02915432	phase1b/2	AGC	all	76	toripalimab	CPS/TMB	CPS: IHC staining with an antihuman PD-L1 monoclonal antibody SP142; TMB: WES; A cut-off of the top 20% of the TMB (12 mutations/Mb) in this study was selected as defining a tumor as TMB-H. Patients with TMB < 12 mutations/Mb were defined as TMB-L	Patients with TMB-H (*n* = 12) had responded significantly better than patients with TMB-L (*n* = 42) (ORR 33.3% versus 7.1%, *p* = 0.017). The TMB-H group showed a numerically longer but not statistically significant PFS than TMB-L group, 2.5 versus 1.9 months, HR = 0.51 (95% CI 0.26–1.02), *p* = 0.055. The TMB-H group showed significant survival advantage in OS than the TMB-L group, 14.6 versus 4.0 months, HR = 0.48 (95% CI 0.24–0.96), *p* = 0.038 [31]
neoadjuvant theraoy	NCT03878472	phase 2	cT4a/bN+ GC	all	25	camrelizumab	CPS; MSI status; TMB; other potential biomarkers	CPS: PD-L1 IHC 22C3 pharmDx kit (Dako); TMB: WES; MSI status: WES; other potential biomarkers: WES, transcriptome sequencing, and T cell receptor (TCR) sequencing	MSI-H patients showed a 100% (3/3) MPR (major pathological response); CPS ≥ 1 patients showed a 100% (3/3) MPR; pathological responses correlate significantly with MSI status, PD-L1 expression, and TMB. Multi-omics analysis identified several potential biomarkers for pathological responses, including RREB1 and SSPO mutations, immune-related signatures, and a peripheral T cell expansion score [32]
	NCT03631615	phase 2	locally advanced GC or GEJC	all	36	camrelizumab	CPS;TMB	CPS: IHC; TMB: whole-exome sequencing	pCR: CPS ≥ 1 vs. no: 44.4% vs. 38.5%; CPS ≥ 5 vs. no: 37.5% vs. 42.9%; CPS ≥ 10 vs. no: 28.6% vs. 46.7%; TMB in total population ≥ median vs. <median: 60.0% vs. 13.3% [33]
	NCT04065282	early results of a phase 2 study	locally advanced, resectable GC or GEJC	patients with resectable G/GEJ adenocarcinoma stage cT3-4NanyM0	45	sintilimab	CPS;MMR;EBV	CPS: IHC 22C3 pharmDx; MMR: IHC; EBV: IHC	In the CPS < 1 subgroup (n = 11), the pCR and MPR rates were 9.1% and 27.3%, respectively. In the 21 patients with CPS ≥ 1, the pCR and MPR rates were 28.6% and 57.1%, respectively. Among the 11 patients with CPS ≥ 5, the pCR and MPR rates were 27.3% and 54.5%, respectively. Among the six patients with CPS ≥ 10, the pCR and MPR rates were 33.3% and 50.0%, respectively. Two (5.6%) patients had dMMR, and one of them, with high PD-L1 expression (CPS = 68), achieved pCR, while the other patient, whose PD-L1 expression is unknown, had a non-major pathological response (TRG2). Among the two (5.6%) patients with EBV-positive status, CPS values of 10 and 0 were found, respectively, and none of them achieved a major pathological response [34]
	NCT02730546	phase 1b/2	resectable GEJC	All	31	pembrolizumab in combination with neoadjuvant chemoradiation and adjuvant pembrolizumab monotherapy	CPS; PD-L1-expressing EVs	IHC staining was performed for PD-L1 CPS and TPS with the 22C3 antibody (Dako). PD-L1-expressing EVs were measured by nanoscale flow cytometry	Patients with CPS ≥ 10 had a significantly higher pCR rate than those with PD-L1 low expression (50.0% [4/8] vs. 13.6% [3/22]; *p* = 0.046). Patients with high PD-L1 expression also experienced longer PFS and OS than propensity-score-matched patients. Among trial patients with PD-L1 CPS < 10, an elevated plasma level of PD-L1-expressing EVs was significantly associated with higher pCR [35]
	NEONIPIGA	phase 2	localized dMMR/MSI-H GC or GEJC	dMMR/MSI-H patients	32	neoadjuvant nivolumab plus ipilimumab and adjuvant nivolumab	MSI	MSI: IHC/PCR	pCR: 59% pathological complete response [36]
second-line/third-line	KEYNOTE-158	phase 2	MSI-H/dMMR GC	dMMR/MSI-H patients	51	pembrolizumab	MSI	IHC/PCR(either the five mononucleotide loci (BAT25,BAT26, NR21, NR24, Mono27) or the five mixed mononucleotide and dinucleotide loci (BAT25, BAT26, Di 5S346,Di 2S123, Di 17S250))	ORR: 31%; mOS: 11 months [37]
first-line or second-line setting	EPOC1706	phase 2	AGC	All	29	lenvatinib + pembrolizumab	CPS;TMB	CPS:PD-L1 IHC 22C3; TMB was measured from the extracted DNA from archival tumor samples using the Oncomine Tumor Mutation Load Assay	TMB ≥ 10 OR: 82% vs. TMB < 10 OR:60%; CPS ≥ 10 ORR: 100%; CPS ≥ 1 ORR: 84%; CPS < 1 ORR: 40% [38]
second-line/third-line	NCT#02589496	phase 2	metastatic GC	all	61	pembrolizumab	CPS/MSI status/EBV/ctDNA	CPS: IHC 22C3 assay; MSI status: WES; EBV: in situ hybridization/WES; ctDNA: a commercially available 73-gene sequencing panel	ORR was 50.0% in PD-L1(+) gastric cancer, 85.7% in MSI-H gastric cancer, and 100% in EBV(+) gastric cancer; ctDNA mutational load score correlated well with clinical response to pembrolizumab [39]
exploratory analysis	exploratory analysis from KEYNOTE-061	phase 3	advanced GC or GEJC	All	420	pembrolizumab	tTMB	tTMB was measured using WES and the FoundationOne^®^CDx (Foundation Medicine, Cambridge, MA)	tTMB ≥ 175 mut/exome: ORR: 30 (Pembrolizumab) vs. 11 (Paclitaxel); OS: 16.4 (Pembrolizumab) vs. 8.1 (Paclitaxel) [40]
	exploratory analysis of the KEYNOTE-062 trial	phase 3	AGC	All	306	pembrolizumab	TMB	TMB was assessed by next-generation sequencing using the FoundationOne CDx; MSI/DNA mismatch repair across five mononucleotide repeat markers (NR21, NR24, BAT25, BAT26, and MONO27) was assessed using DNA extracted from formalin-fixed paraffin-embedded tumor samples and blood (normal control) using the MSI Analysis System, version 1.2 (Promega, Madison, WI, USA)	For pembrolizumab monotherapy compared with chemotherapy, patients in the TMB ≥ 10 mut/Mb subgroup (n = 35) had greater ORR (55.6% vs. 41.2%), PFS (median, 11.1 vs. 7.0 months; HR, 0.52; 95% CI: 0.24–1.13), and OS (median, 31.6 vs. 13.4 months; HR, 0.34; 95% CI: 0.14–0.82) benefit than did patients in the TMB < 10 mut/Mb subgroup [n = 171; ORR, 6.7% vs. 47.6%; median PFS, 2.6 vs. 7.1 months (HR, 1.73; 95% CI: 1.26–2.38); median OS, 7.5 vs. 12.6 months (HR, 1.41; 95% CI: 1.02–1.95)]. After patients with MSI-H tumors were excluded, the positive association between TMB and objective response remained in the pembrolizumab monotherapy group with statistical significance (one-sided *p* = 0.001); however, associations with PFS and OS were no longer significant (one-sided *p* > 0.05) [41]
Prospective study	PMID: 32134806	prospective observational study	stage-IV EBVaGC	stage-IV EBVaGC	9	ICIs	EBV;CPS	EBV status was evaluated by chromogenic EBV-encoded RNA in situ hybridization (Leica Biosystems); CPS: IHC 22C3 assay	33.3% and 55.6% EBVaGC patients showed PR and SD after immunotherapy, all of the patients who showed PR had a positive PD-L1 expression [42]
	NCT03755440	phase 2	metastatic GC	EBER-positive	6	camrelizumab	EBV	EBV:ISH	None of the six EBV-positive mGC patients contracted CR or PR [43]
	DELIVER (Japan Clinical Cancer Research Organisation GC-08) trial	prospective observational study	AGC	all	439	nivolumab	soluble forms of programmed cell deathe1 (sPD-1), PD ligand 1 (sPD-L1) and cytotoxic T lymphocyte-associated proteine4 (sCTLA4)	Plasma levels of sPD-1, sPD-L1 and sCTLA-4 were measured with the use of a fully automated immunoassay system (HISCL, Sysmex)	Higher plasma levels of sPD-1, sPD-L1 and sCTLA-4 were significantly associated with shorter OS, whereas only higher sPD-L1 levels was significantly associated with shorter progression-free survival [44]
Retrospective study	PMID: 35986342	a retrospective analysis	AGC	All	77	ICI	*Helicobacter pylori*	The diagnostic methods for *H. pylori* infection include the 13C-urea breath test (13C-UBT), *H. pylori* stool antigen (HpSA) test and histopathology	Compared with the *H. pylori*-negative group, patients in the *H. pylori*-positive group had a higher risk of nonclinical response to anti-PD-1 antibody, with an OR of 2.91 (95% CI: 1.13–7.50). Patients in the *H. pylori*-negative group had a longer OS and PFS than those in the positive group, with an estimated median OS of 17.5 months vs. 6.2 months (HR = 2.85, 95% CI: 1.70–4.78; *p* = 0.021) and a median PFS of 8.4 months vs. 2.7 months (HR = 3.11, 95% CI: 1.96–5.07, *p* = 0.008). Multivariate analysis indicated that *H. pylori* infection was independently associated with PFS (HR = 1.90, 95% CI: 1.10–3.30; *p* = 0.022) [45]
	PMID: 36092315	a retrospective cohort study	stage IV AGC	CPS PD-L1 negative	26	ICI	TILs	The level of tumor-infiltrating lymphocytes (TILs) was measured by multiplex immunofluorescence (mIF) among these patients	Intertumoral CD8+ T cells were obviously increased in CPS PD-L1 negative patients who responded to chemoimmunotherapy, compared with patients who did not respond (*p* = 0.011). And higher level of CD8+ TILs was demonstrated to associate with better PFS in CPS PD-L1-negative patients treated with chemoimmunotherapy (HR =23.70, 95% CI: 1.15–488.30, *p* = 0.04) [46]

AGC: advanced gastric cancer; CPS: combined positive score; ctDNA: circulating tumor DNA; EBV: Epstein–Barr virus; EBVaGC: EBV-associated gastric cancer; EVs: Extracellular vesicles; GEJC: gastroesophageal junction cancer; HER2: human epidermal growth factor receptor 2; HR: hazard ratio; ICI: immune checkpoint inhibitor; IHC: immunohistochemistry; ISH: in situ hybridization; MPR: major pathological response; MSI: microsatellite unstable; MSI-H: high microsatellite instability; NGS: next-generation sequencing; NLR: neutrophil-to-lymphocyte ratio; ORR: overall response rate; OS: overall survival; PD-L1: programmed death ligand-1; PFS: progression-free survival; pCR: pathologic complete response; PR: partial response; SD: stable disease; TILs: tumor-infiltrating lymphocytes; TMB: tumor mutation burden; TPS: tumor proportion score; tTMB: tissue tumor mutation burden; WES: whole-exome sequencing.

## References

[1] Sung H., Ferlay J., Siegel R.L., Laversanne M., Soerjomataram I., Jemal A., Bray F. (2021). Global Cancer Statistics 2020: GLOBOCAN Estimates of Incidence and Mortality Worldwide for 36 Cancers in 185 Countries. CA Cancer J. Clin..

[2] Joshi S.S., Badgwell B.D. (2021). Current treatment and recent progress in gastric cancer. CA A Cancer J. Clin..

[3] Cancer Genome Atlas Research Network (2014). Comprehensive molecular characterization of gastric adenocarcinoma. Nature.

[4] Cristescu R., Lee J., Nebozhyn M., Kim K.-M., Ting J.C., Wong S.S., Liu J., Yue Y.G., Wang J., Yu K. (2015). Molecular analysis of gastric cancer identifies subtypes associated with distinct clinical outcomes. Nat. Med..

[5] Meric-Bernstam F., Johnson A.M., Dumbrava E.E.I., Raghav K., Balaji K., Bhatt M., Murthy R.K., Rodon J., Piha-Paul S.A. (2019). Advances in HER2-Targeted Therapy: Novel Agents and Opportunities Beyond Breast and Gastric Cancer. Clin. Cancer Res..

[6] Moehler M., Högner A., Wagner A.D., Obermannova R., Alsina M., Thuss-Patience P., van Laarhoven H., Smyth E. (2022). Recent progress and current challenges of immunotherapy in advanced/metastatic esophagogastric adenocarcinoma. Eur. J. Cancer.

[7] Lefler D.S., Snook A.E., Bashir B. (2022). Immune checkpoint inhibitors in luminal gastrointestinal malignancies: Going beyond MSI-H/dMMR, TMB and PD-L1. Immunotherapy.

[8] Lengyel C.G., Hussain S., Trapani D., El Bairi K., Altuna S.C., Seeber A., Odhiambo A., Habeeb B.S., Seid F. (2021). The Emerging Role of Liquid Biopsy in Gastric Cancer. J. Clin. Med..

[9] Huang Y.-K., Wang M., Sun Y., Di Costanzo N., Mitchell C., Achuthan A., Hamilton J.A., Busuttil R.A., Boussioutas A. (2019). Macrophage spatial heterogeneity in gastric cancer defined by multiplex immunohistochemistry. Nat. Commun..

[10] Hoft S.G., Pherson M.D., Di Paolo R.J. (2022). Discovering Immune-Mediated Mechanisms of Gastric Carcinogenesis Through Single-Cell RNA Sequencing. Front. Immunol..

[11] Hou W., Zhou X., Yi C., Zhu H. (2021). Immune Check Point Inhibitors and Immune-Related Adverse Events in Small Cell Lung Cancer. Front. Oncol..

[12] Bagchi S., Yuan R., Engleman E.G. (2021). Immune Checkpoint Inhibitors for the Treatment of Cancer: Clinical Impact and Mechanisms of Response and Resistance. Annu. Rev. Pathol. Mech. Dis..

[13] Yeong J., Lum H.Y.J., Teo C.B., Tan B.K.J., Chan Y.H., Tay R.Y.K., Choo J.R., Jeyasekharan A.D., Miow Q.H., Loo L.H. (2022). Choice of PD-L1 immunohistochemistry assay influences clinical eligibility for gastric cancer immunotherapy. Gastric Cancer.

[14] Muro K., Chung H.C., Shankaran V., Geva R., Catenacci D., Gupta S., Eder J.P., Golan T., Le D.T., Burtness B. (2016). Pembrolizumab for patients with PD-L1-positive advanced gastric cancer (KEYNOTE-012): A multicentre, open-label, phase 1b trial. Lancet Oncol..

[15] Fuchs C.S., Doi T., Jang R.W., Muro K., Satoh T., Machado M., Sun W., Jalal S.I., Shah M.A., Metges J.P. (2018). Safety and Efficacy of Pembrolizumab Monotherapy in Patients With Previously Treated Advanced Gastric and Gastroesophageal Junction Cancer: Phase 2 Clinical KEYNOTE-059 Trial. JAMA Oncol..

[16] Janjigian Y.Y., Bendell J., Calvo E., Kim J.W., Ascierto P.A., Sharma P., Ott P.A., Peltola K., Jaeger D., Evans J. (2018). CheckMate-032 Study: Efficacy and Safety of Nivolumab and Nivolumab Plus Ipilimumab in Patients With Metastatic Esophagogastric Cancer. J. Clin. Oncol..

[17] Lei M., Siemers N.O., Pandya D., Chang H., Sanchez T., Harbison C., Szabo P.M., Janjigian Y., Ott P.A., Sharma P. (2021). Analyses of PD-L1 and Inflammatory Gene Expression Association with Efficacy of Nivolumab ± Ipilimumab in Gastric Cancer/Gastroesophageal Junction Cancer. Clin. Cancer Res..

[18] Kang Y.-K., Boku N., Satoh T., Ryu M.-H., Chao Y., Kato K., Chung H.C., Chen J.-S., Muro K., Kang W.K. (2017). Nivolumab in patients with advanced gastric or gastro-oesophageal junction cancer refractory to, or intolerant of, at least two previous chemotherapy regimens (ONO-4538-12, ATTRACTION-2): A randomised, double-blind, placebo-controlled, phase 3 trial. Lancet.

[19] Kim J.H., Ryu M.-H., Park Y.S., Ma J., Lee S.Y., Kim D., Kang Y.-K. (2022). Predictive biomarkers for the efficacy of nivolumab as ≥ 3rd-line therapy in patients with advanced gastric cancer: A subset analysis of ATTRACTION-2 phase III trial. BMC Cancer.

[20] Bang Y.-J., Ruiz E., Van Cutsem E., Lee K.-W., Wyrwicz L., Schenker M., Alsina M., Ryu M.-H., Chung H.-C., Evesque L. (2018). Phase III, randomised trial of avelumab versus physician’s choice of chemotherapy as third-line treatment of patients with advanced gastric or gastro-oesophageal junction cancer: Primary analysis of JAVELIN Gastric 300. Ann. Oncol..

[21] Moehler M., Dvorkin M., Boku N., Özgüroğlu M., Ryu M.-H., Muntean A.S., Lonardi S., Nechaeva M., Bragagnoli A.C., Coşkun H.S. (2021). Phase III Trial of Avelumab Maintenance After First-Line Induction Chemotherapy Versus Continuation of Chemotherapy in Patients With Gastric Cancers: Results From JAVELIN Gastric 100. J. Clin. Oncol..

[22] Shitara K., Özgüroğlu M., Bang Y.-J., Di Bartolomeo M., Mandalà M., Ryu M.-H., Fornaro L., Olesiński T., Caglevic C., Muro K. (2018). Pembrolizumab versus paclitaxel for previously treated, advanced gastric or gastro-oesophageal junction cancer (KEYNOTE-061): A randomised, open-label, controlled, phase 3 trial. Lancet.

[23] Nakajima T.E., Kadowaki S., Minashi K., Nishina T., Yamanaka T., Hayashi Y., Izawa N., Muro K., Hironaka S., Kajiwara T. (2021). Multicenter Phase I/II Study of Nivolumab Combined with Paclitaxel Plus Ramucirumab as Second-line Treatment in Patients with Advanced Gastric Cancer. Clin. Cancer Res..

[24] Thuss-Patience P.C., Högner A., Goekkurt E., Stahl M., Kretzschmar A., Schädlich B., Goetze T.O., Stocker G., Reichardt P., Kullmann F. (2022). Ramucirumab, avelumab, and paclitaxel (RAP) as second-line treatment in gastro-esophageal adenocarcinoma, a phase II trial of the Arbeitsgemeinschaft Internistische Onkologie (AIO). J. Clin. Oncol..

[25] Catenacci D.V.T., Kang Y.K., Park H., Uronis H.E., Lee K.W., Ng M.C.H., Enzinger P.C., Park S.H., Gold P.J., Lacy J. (2020). Margetuximab plus pembrolizumab in patients with previously treated, HER2-positive gastro-oesophageal adenocarcinoma (CP-MGAH22-05): A single-arm, phase 1b-2 trial. Lancet Oncol..

[26] Janjigian Y.Y., Shitara K., Moehler M., Garrido M., Salman P., Shen L., Wyrwicz L., Yamaguchi K., Skoczylas T., Bragagnoli A.C. (2021). First-line nivolumab plus chemotherapy versus chemotherapy alone for advanced gastric, gastro-oesophageal junction, and oesophageal adenocarcinoma (CheckMate 649): A randomised, open-label, phase 3 trial. Lancet.

[27] Xu J., Jiang H., Pan Y., Gu K., Cang S., Han L., Shu Y., Li J., Zhao J., Pan H. (2021). LBA53 Sintilimab plus chemotherapy (chemo) versus chemo as first-line treatment for advanced gastric or gastroesophageal junction (G/GEJ) adenocarcinoma (ORIENT-16): First results of a randomized, double-blind, phase III study. Ann. Oncol..

[28] Shitara K., Van Cutsem E., Bang Y.-J., Fuchs C., Wyrwicz L., Lee K.-W., Kudaba I., Garrido M., Chung H.C., Lee J. (2020). Efficacy and Safety of Pembrolizumab or Pembrolizumab Plus Chemotherapy vs Chemotherapy Alone for Patients With First-line, Advanced Gastric Cancer: The KEYNOTE-062 Phase 3 Randomized Clinical Trial. JAMA Oncol..

[29] Kang Y.-K., Chen L.-T., Ryu M.-H., Oh D.-Y., Oh S.C., Chung H.C., Lee K.-W., Omori T., Shitara K., Sakuramoto S. (2022). Nivolumab plus chemotherapy versus placebo plus chemotherapy in patients with HER2-negative, untreated, unresectable advanced or recurrent gastric or gastro-oesophageal junction cancer (ATTRACTION-4): A randomised, multicentre, double-blind, placebo-controlled, phase 3 trial. Lancet Oncol..

[30] Yamaguchi K., Minashi K., Sakai D., Nishina T., Omuro Y., Tsuda M., Iwagami S., Kawakami H., Esaki T., Sugimoto N. (2022). Phase IIb study of pembrolizumab combined with S-1 + oxaliplatin or S-1 + cisplatin as first-line chemotherapy for gastric cancer. Cancer Sci..

[31] Wang F.H., Wei X., Xu N., Shen L., Dai G., Yuan X., Chen Y., Yang S., Shi J., Hu X. (2019). Safety, efficacy and tumor mutational burden as a biomarker of overall survival benefit in chemo-refractory gastric cancer treated with toripalimab, a PD-1 antibody in phase Ib/II clinical trial NCT02915432. Ann. Oncol..

[32] Li S., Yu W., Xie F., Luo H., Liu Z., Lv W., Shi D., Yu D., Gao P., Chen C. (2023). Neoadjuvant therapy with immune checkpoint blockade, antiangiogenesis, and chemotherapy for locally advanced gastric cancer. Nat. Commun..

[33] Tang Z., Wang Y., Liu D., Wang X., Xu C., Yu Y., Cui Y., Tang C., Li Q., Sun J. (2022). The Neo-PLANET phase II trial of neoadjuvant camrelizumab plus concurrent chemoradiotherapy in locally advanced adenocarcinoma of stomach or gastroesophageal junction. Nat. Commun..

[34] Jiang H., Yu X., Li N., Kong M., Ma Z., Zhou D., Wang W., Wang H., Wang H., He K. (2022). Efficacy and safety of neoadjuvant sintilimab, oxaliplatin and capecitabine in patients with locally advanced, resectable gastric or gastroesophageal junction adenocarcinoma: Early results of a phase 2 study. J. Immunother. Cancer.

[35] Zhu M., Chen C., Foster N.R., Hartley C., Mounajjed T., Salomao M.A., Fruth B.F., Beamer S.E., Kim Y., Harrington S.M. (2022). Pembrolizumab in Combination with Neoadjuvant Chemoradiotherapy for Patients with Resectable Adenocarcinoma of the Gastroesophageal Junction. Clin. Cancer Res..

[36] André T., Tougeron D., Piessen G., de la Fouchardière C., Louvet C., Adenis A., Jary M., Tournigand C., Aparicio T., Desrame J. (2023). Neoadjuvant Nivolumab Plus Ipilimumab and Adjuvant Nivolumab in Localized Deficient Mismatch Repair/Microsatellite Instability–High Gastric or Esophagogastric Junction Adenocarcinoma: The GERCOR NEONIPIGA Phase II Study. J. Clin. Oncol..

[37] Maio M., Ascierto P., Manzyuk L., Motola-Kuba D., Penel N., Cassier P., Bariani G., Acosta A.D.J., Doi T., Longo F. (2022). Pembrolizumab in microsatellite instability high or mismatch repair deficient cancers: Updated analysis from the phase II KEYNOTE-158 study. Ann. Oncol..

[38] Kawazoe A., Fukuoka S., Nakamura Y., Kuboki Y., Wakabayashi M., Nomura S., Mikamoto Y., Shima H., Fujishiro N., Higuchi T. (2020). Lenvatinib plus pembrolizumab in patients with advanced gastric cancer in the first-line or second-line setting (EPOC1706): An open-label, single-arm, phase 2 trial. Lancet Oncol..

[39] Kim S.T., Cristescu R., Bass A.J., Kim K.-M., Odegaard J.I., Kim K., Liu X.Q., Sher X., Jung H., Lee M. (2018). Comprehensive molecular characterization of clinical responses to PD-1 inhibition in metastatic gastric cancer. Nat. Med..

[40] Shitara K., Di Bartolomeo M., Mandala M., Ryu M.-H., Caglevic C., Olesinski T., Chung H.C., Muro K., Goekkurt E., McDermott R.S. (2023). Association between gene expression signatures and clinical outcomes of pembrolizumab versus paclitaxel in advanced gastric cancer: Exploratory analysis from the randomized, controlled, phase III KEYNOTE-061 trial. J. Immunother. Cancer.

[41] Lee K.W., Van Cutsem E., Bang Y.J., Fuchs C.S., Kudaba I., Garrido M., Chung H.C., Lee J., Castro H.R., Chao J. (2022). Association of Tumor Mutational Burden with Efficacy of Pembrolizumab±Chemotherapy as First-Line Therapy for Gastric Cancer in the Phase III KEYNOTE-062 Study. Clin. Cancer Res..

[42] Xie T., Liu Y., Zhang Z., Zhang X., Gong J., Qi C., Li J., Shen L., Peng Z. (2020). Positive Status of Epstein-Barr Virus as a Biomarker for Gastric Cancer Immunotherapy: A Prospective Observational Study. J. Immunother..

[43] Sun Y.-T., Guan W.-L., Zhao Q., Wang D.-S., Lu S.-X., He C.-Y., Chen S.-Z., Wang F.-H., Li Y.-H., Zhou Z.-W. (2021). PD-1 antibody camrelizumab for Epstein-Barr virus-positive metastatic gastric cancer: A single-arm, open-label, phase 2 trial. Am. J. Cancer Res..

[44] Kawakami H., Sunakawa Y., Inoue E., Matoba R., Noda K., Sato T., Suminaka C., Yamaki M., Sakamoto Y., Kawabata R. (2023). Soluble programmed cell death ligand 1 predicts prognosis for gastric cancer patients treated with nivolumab: Blood-based biomarker analysis for the DELIVER trial. Eur. J. Cancer.

[45] Che H., Xiong Q., Ma J., Chen S., Wu H., Xu H., Hou B. (2022). Association of *Helicobacter pylori* infection with survival outcomes in advanced gastric cancer patients treated with immune checkpoint inhibitors. BMC Cancer.

[46] Tong G., Zhu M., Chen Y., Wang S., Cheng B., Wang S., Liao W. (2022). Intratumoral CD8(+) T cells as a potential positive predictor of chemoimmunotherapy response in PD-L1-negative advanced gastric cancer patients: A retrospective cohort study. J. Gastrointest. Oncol..

[47] Fuchs C.S., Özgüroğlu M., Bang Y.-J., Di Bartolomeo M., Mandala M., Ryu M.-H., Fornaro L., Olesinski T., Caglevic C., Chung H.C. (2021). Pembrolizumab versus paclitaxel for previously treated PD-L1-positive advanced gastric or gastroesophageal junction cancer: 2-year update of the randomized phase 3 KEYNOTE-061 trial. Gastric Cancer.

[48] Shitara K., Ajani J.A., Moehler M., Garrido M., Gallardo C., Shen L., Yamaguchi K., Wyrwicz L., Skoczylas T., Bragagnoli A.C. (2022). Nivolumab plus chemotherapy or ipilimumab in gastro-oesophageal cancer. Nature.

[49] Zhao J.J., Yap D.W.T., Chan Y.H., Tan B.K.J., Teo C.B., Syn N.L., Smyth E.C., Soon Y.Y., Sundar R. (2022). Low Programmed Death-Ligand 1–Expressing Subgroup Outcomes of First-Line Immune Checkpoint Inhibitors in Gastric or Esophageal Adenocarcinoma. J. Clin. Oncol..

[50] Yoon H.H., Jin Z., Kour O., Kankeu Fonkoua L.A., Shitara K., Gibson M.K., Prokop L.J., Moehler M., Kang Y.K., Shi Q. (2022). Association of PD-L1 Expression and Other Variables With Benefit From Immune Checkpoint Inhibition in Advanced Gastroesophageal Cancer: Systematic Review and Meta-analysis of 17 Phase 3 Randomized Clinical Trials. JAMA Oncol..

[51] Dubois M., Liscia N., Brunetti O., Ziranu P., Lai E., Argentiero A., Mazza E., Cascinu S., Silvestris N., Casadei-Gardini A. (2022). The role of immune checkpoint inhibitors in the treatment sequence of advanced gastric or gastro-esophageal junction cancer: A systematic review and meta-analysis of randomized trials. Crit. Rev. Oncol..

[52] Xie T., Zhang Z., Zhang X., Qi C., Shen L., Peng Z. (2021). Appropriate PD-L1 Cutoff Value for Gastric Cancer Immunotherapy: A Systematic Review and Meta-Analysis. Front. Oncol..

[53] Wainberg Z.A., Fuchs C.S., Tabernero J., Shitara K., Muro K., Van Cutsem E., Bang Y.-J., Chung H.C., Yamaguchi K., Varga E. (2021). Efficacy of Pembrolizumab Monotherapy for Advanced Gastric/Gastroesophageal Junction Cancer with Programmed Death Ligand 1 Combined Positive Score ≥10. Clin. Cancer Res..

[54] Hagi T., Kurokawa Y., Kawabata R., Omori T., Matsuyama J., Fujitani K., Hirao M., Akamaru Y., Takahashi T., Yamasaki M. (2020). Multicentre biomarker cohort study on the efficacy of nivolumab treatment for gastric cancer. Br. J. Cancer.

[55] Schoemig-Markiefka B., Eschbach J., Scheel A.H., Pamuk A., Rueschoff J., Zander T., Buettner R., Schroeder W., Bruns C.J., Loeser H. (2021). Optimized PD-L1 scoring of gastric cancer. Gastric Cancer.

[56] Yamashita K., Iwatsuki M., Harada K., Eto K., Hiyoshi Y., Ishimoto T., Nagai Y., Iwagami S., Miyamoto Y., Yoshida N. (2020). Prognostic impacts of the combined positive score and the tumor proportion score for programmed death ligand-1 expression by double immunohistochemical staining in patients with advanced gastric cancer. Gastric Cancer.

[57] Davis A.A., Patel V.G. (2019). The role of PD-L1 expression as a predictive biomarker: An analysis of all US Food and Drug Administration (FDA) approvals of immune checkpoint inhibitors. J. Immunother. Cancer.

[58] Doroshow D.B., Bhalla S., Beasley M.B., Sholl L.M., Kerr K.M., Gnjatic S., Wistuba I.I., Rimm D.L., Tsao M.S., Hirsch F.R. (2021). PD-L1 as a biomarker of response to immune-checkpoint inhibitors. Nat. Rev. Clin. Oncol..

[59] Venkatasamy A., Guerin E., Reichardt W., Devignot V., Chenard M.P., Miguet L., Romain B., Jung A.C., Gross I., Gaiddon C. (2022). Morpho-functional analysis of patient-derived xenografts reveals differential impact of gastric cancer and chemotherapy on the tumor ecosystem, affecting immune check point, metabolism, and sarcopenia. Gastric Cancer.

[60] Marcus L., Lemery S.J., Keegan P., Pazdur R. (2019). FDA Approval Summary: Pembrolizumab for the Treatment of Microsatellite Instability-High Solid Tumors. Clin. Cancer Res..

[61] Dudley J.C., Lin M.-T., Le D.T., Eshleman J.R. (2016). Microsatellite Instability as a Biomarker for PD-1 Blockade. Clin. Cancer Res..

[62] Polom K., Marrelli D., Roviello G., Pascale V., Voglino C., Rho H., Marini M., Macchiarelli R., Roviello F. (2017). Molecular key to understand the gastric cancer biology in elderly patients—The role of microsatellite instability. J. Surg. Oncol..

[63] Chao J., Fuchs C.S., Shitara K., Tabernero J., Muro K., Van Cutsem E., Bang Y.J., De Vita F., Landers G., Yen C.J. (2021). Assessment of Pembrolizumab Therapy for the Treatment of Microsatellite Instability-High Gastric or Gastroesophageal Junction Cancer Among Patients in the KEYNOTE-059, KEYNOTE-061, and KEYNOTE-062 Clinical Trials. JAMA Oncol..

[64] Pietrantonio F., Randon G., Di Bartolomeo M., Luciani A., Chao J., Smyth E., Petrelli F. (2021). Predictive role of microsatellite instability for PD-1 blockade in patients with advanced gastric cancer: A meta-analysis of randomized clinical trials. ESMO Open.

[65] Puliga E., Corso S., Pietrantonio F., Giordano S. (2021). Microsatellite instability in Gastric Cancer: Between lights and shadows. Cancer Treat. Rev..

[66] Hou W., Yi C., Zhu H. (2022). Predictive biomarkers of colon cancer immunotherapy: Present and future. Front. Immunol..

[67] Yang Y., Shi Z., Bai R., Hu W. (2021). Heterogeneity of MSI-H gastric cancer identifies a subtype with worse survival. J. Med. Genet..

[68] Hause R.J., Pritchard C.C., Shendure J., Salipante S.J. (2016). Classification and characterization of microsatellite instability across 18 cancer types. Nat. Med..

[69] Kwon M., An M., Klempner S.J., Lee H., Kim K.-M., Sa J.K., Cho H.J., Hong J.Y., Lee T., Min Y.W. (2021). Determinants of Response and Intrinsic Resistance to PD-1 Blockade in Microsatellite Instability–High Gastric Cancer. Cancer Discov..

[70] Addeo A., Friedlaender A., Banna G.L., Weiss G.J. (2021). TMB or not TMB as a biomarker: That is the question. Crit. Rev. Oncol..

[71] McGrail D., Pilié P., Rashid N., Voorwerk L., Slagter M., Kok M., Jonasch E., Khasraw M., Heimberger A., Lim B. (2021). High tumor mutation burden fails to predict immune checkpoint blockade response across all cancer types. Ann. Oncol..

[72] Jardim D.L., Goodman A., de Melo Gagliato D., Kurzrock R. (2021). The Challenges of Tumor Mutational Burden as an Immunotherapy Biomarker. Cancer Cell.

[73] Shitara K., Özgüroğlu M., Bang Y.-J., Di Bartolomeo M., Mandalà M., Ryu M.-H., Caglevic C., Chung H., Muro K., Van Cutsem E. (2021). Molecular determinants of clinical outcomes with pembrolizumab versus paclitaxel in a randomized, open-label, phase III trial in patients with gastroesophageal adenocarcinoma. Ann. Oncol..

[74] Alborelli I., Leonards K., Rothschild S.I., Leuenberger L.P., Savic Prince S., Mertz K.D., Poechtrager S., Buess M., Zippelius A., Läubli H. (2020). Tumor mutational burden assessed by targeted NGS predicts clinical benefit from immune checkpoint inhibitors in non-small cell lung cancer. J. Pathol..

[75] Chida K., Kawazoe A., Kawazu M., Suzuki T., Nakamura Y., Nakatsura T., Kuwata T., Ueno T., Kuboki Y., Kotani D. (2021). A Low Tumor Mutational Burden and PTEN Mutations Are Predictors of a Negative Response to PD-1 Blockade in MSI-H/dMMR Gastrointestinal Tumors. Clin. Cancer Res..

[76] Wang Z., Zhao X., Gao C., Gong J., Wang X., Gao J., Li Z., Wang J., Yang B., Wang L. (2020). Plasma-based microsatellite instability detection strategy to guide immune checkpoint blockade treatment. J. Immunother. Cancer.

[77] Kanda T., Yajima M., Ikuta K. (2019). Epstein-Barr virus strain variation and cancer. Cancer Sci..

[78] De Re V., Brisotto G., Repetto O., De Zorzi M., Caggiari L., Zanussi S., Alessandrini L., Canzonieri V., Miolo G., Puglisi F. (2020). Overview of Epstein–Barr-Virus-Associated Gastric Cancer Correlated with Prognostic Classification and Development of Therapeutic Options. Int. J. Mol. Sci..

[79] Kim S.Y., Park C., Kim H.-J., Park J., Hwang J., Kim J.-I., Choi M.G., Kim S., Kim K.-M., Kang M.-S. (2015). Deregulation of Immune Response Genes in Patients With Epstein-Barr Virus-Associated Gastric Cancer and Outcomes. Gastroenterology.

[80] Derks S., Liao X., Chiaravalli A.M., Xu X., Camargo M.C., Solcia E., Sessa F., Fleitas T., Freeman G.J., Rodig S.J. (2016). Abundant PD-L1 expression in Epstein-Barr Virus-infected gastric cancers. Oncotarget.

[81] Salnikov M., Prusinkiewicz M.A., Lin S., Ghasemi F., Cecchini M.J., Mymryk J.S. (2023). Tumor-Infiltrating T Cells in EBV-Associated Gastric Carcinomas Exhibit High Levels of Multiple Markers of Activation, Effector Gene Expression, and Exhaustion. Viruses.

[82] Nakayama A., Abe H., Kunita A., Saito R., Kanda T., Yamashita H., Seto Y., Ishikawa S., Fukayama M. (2019). Viral loads correlate with upregulation of PD-L1 and worse patient prognosis in Epstein–Barr Virus-associated gastric carcinoma. PLoS ONE.

[83] Sun K., Jia K., Lv H., Wang S.-Q., Wu Y., Lei H., Chen X. (2020). EBV-Positive Gastric Cancer: Current Knowledge and Future Perspectives. Front. Oncol..

[84] Bai Y., Xie T., Wang Z., Tong S., Zhao X., Zhao F., Cai J., Wei X., Peng Z., Shen L. (2022). Efficacy and predictive biomarkers of immunotherapy in Epstein-Barr virus-associated gastric cancer. J. Immunother. Cancer.

[85] Dislich B., Mertz K.D., Gloor B., Langer R. (2022). Interspatial Distribution of Tumor and Immune Cells in Correlation with PD-L1 in Molecular Subtypes of Gastric Cancers. Cancers.

[86] Yu H.-Y., Li C.-P., Huang Y.-H., Hsu S.-J., Wang Y.-P., Hsieh Y.-C., Fang W.-L., Huang K.-H., Li A.F.-Y., Lee R.-C. (2022). Microsatellite Instability, Epstein–Barr Virus, and Programmed Cell Death Ligand 1 as Predictive Markers for Immunotherapy in Gastric Cancer. Cancers.

[87] Ishiba T., Hoffmann A.C., Usher J., Elshimali Y., Sturdevant T., Dang M., Jaimes Y., Tyagi R., Gonzales R., Grino M. (2018). Frequencies and expression levels of programmed death ligand 1 (PD-L1) in circulating tumor RNA (ctRNA) in various cancer types. Biochem. Biophys. Res. Commun..

[88] Jin Y., Chen D.-L., Yang C.-P., Chen X.-X., You J.-Q., Huang J.-S., Shao Y., Zhu D.-Q., Ouyang Y.-M., Luo H.-Y. (2020). The predicting role of circulating tumor DNA landscape in gastric cancer patients treated with immune checkpoint inhibitors. Mol. Cancer.

[89] Zhang M., Qi C., Wang Z., Chen H., Zhao X., Zhang X., Zhou Y., Gao C., Bai Y., Jia S. (2021). Molecular characterization of ctDNA from Chinese patients with advanced gastric adenocarcinoma reveals actionable alterations for targeted and immune therapy. J. Mol. Med..

[90] Yue C., Jiang Y., Li P., Wang Y., Xue J., Li N., Li D., Wang R., Dang Y., Hu Z. (2018). Dynamic change of PD-L1 expression on circulating tumor cells in advanced solid tumor patients undergoing PD-1 blockade therapy. OncoImmunology.

[91] Zhang C., Chong X., Jiang F., Gao J., Chen Y., Jia K., Fan M., Liu X., An J., Li J. (2022). Plasma extracellular vesicle derived protein profile predicting and monitoring immunotherapeutic outcomes of gastric cancer. J. Extracell. Vesicles.

[92] Nikanjam M., Kato S., Kurzrock R. (2022). Liquid biopsy: Current technology and clinical applications. J. Hematol. Oncol..

[93] Lee P.-C., Wu C.-J., Hung Y.-W., Lee C.J., Chi C.-T., Lee I.-C., Yu-Lun K., Chou S.-H., Luo J.-C., Hou M.-C. (2022). Gut microbiota and metabolites associate with outcomes of immune checkpoint inhibitor–treated unresectable hepatocellular carcinoma. J. Immunother. Cancer.

[94] Thomas A.M., Fidelle M., Routy B., Kroemer G., Wargo J.A., Segata N., Zitvogel L. (2023). Gut OncoMicrobiome Signatures (GOMS) as next-generation biomarkers for cancer immunotherapy. Nat. Rev. Clin. Oncol..

[95] Liu D., Zhu J., Ma X., Zhang L., Wu Y., Zhu W., Xing Y., Jia Y., Wang Y. (2021). Transcriptomic and Metabolomic Profiling in *Helicobacter pylori*–Induced Gastric Cancer Identified Prognosis- and Immunotherapy-Relevant Gene Signatures. Front. Cell Dev. Biol..

[96] Deng R., Zheng H., Cai H., Li M., Shi Y., Ding S. (2022). Effects of *Helicobacter pylori* on tumor microenvironment and immunotherapy responses. Front. Immunol..

[97] Oster P., Vaillant L., Riva E., McMillan B., Begka C., Truntzer C., Richard C., Leblond M.M., Messaoudene M., Machremi E. (2021). *Helicobacter pylori* infection has a detrimental impact on the efficacy of cancer immunotherapies. Gut.

[98] Derks S., de Klerk L.K., Xu X., Fleitas T., Liu K.X., Liu Y., Dietlein F., Margolis C., Chiaravalli A.M., Da Silva A.C. (2020). Characterizing diversity in the tumor-immune microenvironment of distinct subclasses of gastroesophageal adenocarcinomas. Ann. Oncol..

[99] Hu X., Wang Z., Wang Q., Chen K., Han Q., Bai S., Du J., Chen W. (2021). Molecular classification reveals the diverse genetic and prognostic features of gastric cancer: A multi-omics consensus ensemble clustering. Biomed. Pharmacother..

[100] Fu M., Huang Y., Peng X., Li X., Luo N., Zhu W., Yang F., Chen Z., Ma S., Zhang Y. (2022). Development of Tumor Mutation Burden-Related Prognostic Model and Novel Biomarker Identification in Stomach Adenocarcinoma. Front. Cell Dev. Biol..

[101] Yuan Q., Deng D., Pan C., Ren J., Wei T., Wu Z., Zhang B., Li S., Yin P., Shang D. (2022). Integration of transcriptomics, proteomics, and metabolomics data to reveal HER2-associated metabolic heterogeneity in gastric cancer with response to immunotherapy and neoadjuvant chemotherapy. Front. Immunol..

[102] Wang K.-W., Wang M.-D., Li Z.-X., Hu B.-S., Wu J.-J., Yuan Z.-D., Wu X.-L., Yuan Q.-F., Yuan F.-L. (2022). An antigen processing and presentation signature for prognostic evaluation and immunotherapy selection in advanced gastric cancer. Front. Immunol..

[103] Shi J., Wu Z., Wu X., Huangfu L., Guo T., Cheng X., Han J., Li Z., Xing X., Ji J. (2023). Characterization of glycometabolism and tumor immune microenvironment for predicting clinical outcomes in gastric cancer. iScience.

[104] Zeng D., Wu J., Luo H., Li Y., Xiao J., Peng J., Ye Z., Zhou R., Yu Y., Wang G. (2021). Tumor microenvironment evaluation promotes precise checkpoint immunotherapy of advanced gastric cancer. J. Immunother. Cancer.

[105] He Y., Wang X. (2020). Identification of molecular features correlating with tumor immunity in gastric cancer by multi-omics data analysis. Ann. Transl. Med..

[106] Chen Y., Sun Z., Chen W., Liu C., Chai R., Ding J., Liu W., Feng X., Zhou J., Shen X. (2021). The Immune Subtypes and Landscape of Gastric Cancer and to Predict Based on the Whole-Slide Images Using Deep Learning. Front. Immunol..

[107] Cheong J.-H., Wang S.C., Park S., Porembka M.R., Christie A.L., Kim H., Kim H.S., Zhu H., Hyung W.J., Noh S.H. (2022). Development and validation of a prognostic and predictive 32-gene signature for gastric cancer. Nat. Commun..

[108] Chuah S., Chew V. (2020). High-dimensional immune-profiling in cancer: Implications for immunotherapy. J. Immunother. Cancer.

[109] Cabrita R., Lauss M., Sanna A., Donia M., Skaarup Larsen M., Mitra S., Johansson I., Phung B., Harbst K., Vallon-Christersson J. (2020). Tertiary lymphoid structures improve immunotherapy and survival in melanoma. Nature.

[110] Paijens S.T., Vledder A., de Bruyn M., Nijman H.W. (2021). Tumor-infiltrating lymphocytes in the immunotherapy era. Cell. Mol. Immunol..

[111] Schumacher T.N., Thommen D.S. (2022). Tertiary lymphoid structures in cancer. Science.

[112] Jiang Q., Tian C., Wu H., Min L., Chen H., Chen L., Liu F., Sun Y. (2022). Tertiary lymphoid structure patterns predicted anti-PD1 therapeutic responses in gastric cancer. Chin. J. Cancer Res..

[113] Xu C., Fang H., Gu Y., Yu K., Wang J., Lin C., Zhang H., Li H., He H., Liu H. (2022). Impact of intratumouralCD96expression on clinical outcome and therapeutic benefit in gastric cancer. Cancer Sci..

[114] He X., Gu Y., Cao Y., Hu B., Fang H., Fei Y., Lv K., Liu X., Wang J., Lin C. (2021). Impact of intratumoural CD73 expression on prognosis and therapeutic response in patients with gastric cancer. Eur. J. Cancer.

[115] Lu S., Stein J.E., Rimm D.L., Wang D.W., Bell J.M., Johnson D.B., Sosman J.A., Schalper K.A., Anders R.A., Wang H. (2019). Comparison of Biomarker Modalities for Predicting Response to PD-1/PD-L1 Checkpoint Blockade: A Systematic Review and Meta-analysis. JAMA Oncol..

[116] Chen Y., Jia K., Sun Y., Zhang C., Li Y., Zhang L., Chen Z., Zhang J., Hu Y., Yuan J. (2022). Predicting response to immunotherapy in gastric cancer via multi-dimensional analyses of the tumour immune microenvironment. Nat. Commun..

[117] Wang Q., Xu R. (2018). Immunotherapy-related adverse events (irAEs): Extraction from FDA drug labels and comparative analysis. JAMIA Open.

[118] Mazzarella L., Giugliano F., Nicolo E., Esposito A., Crimini E., Tini G., Uliano J., Corti C., D’amico P., Aliaga P.T. (2023). Immune-Related Adverse Event Likelihood Score Identifies “Pure” IRAEs Strongly Associated With Outcome in a Phase I–II Trial Population. Oncologist.

[119] Bai R., Li L., Chen X., Chen N., Song W., Zhang Y., Lv Z., Han F., Zhao Y., Li W. (2021). Correlation of Peripheral Blood Parameters and Immune-Related Adverse Events with the Efficacy of Immune Checkpoint Inhibitors. J. Oncol..

[120] Masuda K., Shoji H., Nagashima K., Yamamoto S., Ishikawa M., Imazeki H., Aoki M., Miyamoto T., Hirano H., Honma Y. (2019). Correlation between immune-related adverse events and prognosis in patients with gastric cancer treated with nivolumab. BMC Cancer.

[121] Suematsu H., Kano K., Yamada T., Hashimoto I., Watanabe H., Takahashi K., Watanabe M., Hayashi K., Kaneta Y., Furuta M. (2022). Prognostic Impact of Immune-related Adverse Events in Gastric Cancer Patients Treated With Nivolumab. Anticancer. Res..

[122] Zhang X., Xu S., Wang J., Lv Y., Wang N., Lai R., Sha Z., Zhao Q., Guo Z. (2022). Are anti-PD-1-associated immune related adverse events a harbinger of favorable clinical prognosis in patients with gastric cancer?. BMC Cancer.

[123] Bai R., Chen N., Chen X., Li L., Song W., Li W., Zhao Y., Zhang Y., Han F., Lyu Z. (2021). Analysis of characteristics and predictive factors of immune checkpoint inhibitor-related adverse events. Cancer Biol. Med..

[124] Jiang F., Zhang Z., Chong X., Shen L., Fan M., Liu X., An J., Peng Z., Zhang C. (2022). Extracellular Vesicle-Derived Protein File from Peripheral Blood Predicts Immune-Related Adverse Events in Gastric Cancer Patients Receiving Immunotherapy. Cancers.

